# Intact B-Cell Signaling and Function With Host B-Cells 47 Years After Transplantation for X-SCID

**DOI:** 10.3389/fimmu.2020.00415

**Published:** 2020-03-20

**Authors:** Christin Deal, Timothy J. Thauland, E. Richard Stiehm, Maria I. Garcia-Lloret, Manish J. Butte

**Affiliations:** Division of Immunology, Allergy, and Rheumatology, Department of Pediatrics, University of California, Los Angeles, Los Angeles, CA, United States

**Keywords:** SCID, B cell, transplant, chimerism, IL2RG, IL-21, gamma chain, IVIG

## Abstract

**Introduction:** Severe Combined Immunodeficiency (SCID) is a life-threatening immunodeficiency caused by several pathogenic genetic variants, and it is characterized by profound defects in T-cell numbers and immune function. First performed in the late 1960's, hematopoietic stem cell transplantation remains the standard treatment for most cases of SCID. There is a growing number of post-transplant SCID patients, and it is imperative to assess the long-term outcomes of these patients. We have reported here the longest follow-up of a post-transplant SCID patient who, to our knowledge, bears the first gamma chain (γc) variant to show intact IL-21 signaling.

**Case Presentation:** The patient presented at 5 months of age with recurrent thrush and *Pneumocystis jiroveci* pneumonia. In 1971, at the age of 11 months, he received an unconditioned, matched, related donor transplant comprising whole, unprocessed bone marrow. He is now 48 years old without significant illness and has never required immunoglobulin replacement. He exhibits T-dependent vaccine responses. He does suffer from chronic warts and bacterial infections that have worsened in recent years. We confirmed a known pathogenic variant in the *IL2RG* gene showing a hemizygous variant NM_000206.2:c.675C>A, resulting in p.Ser225Arg. His chimerism studies revealed donor T cells, host B cells, host myeloid cells, and mixed NK cells. Lymphocyte enumeration revealed normal numbers and distribution of B cells. The host B cells carry the pathogenic variant in *IL2RG*, but, when stimulated with IL-21, they demonstrated intact, γc-dependent signaling.

**Conclusions:** Even with host B cells, reconstitution with donor T cells can be sufficient to allow over four decades of survival when B-cell function is intact. Our case demonstrates that satisfactory B-cell function can arise as a consequence of both intact IL-21 signaling due to a hypomorphic γc variant, and close HLA matching with the donor to allow for effective T-cell help.

## Introduction

Severe Combined Immunodeficiency (SCID) is a collection of life-threatening diseases characterized by an absence of functional T cells and requiring treatment with hematopoietic stem cell transplantation (HSCT), gene therapy, or enzyme replacement therapy. At least 70 SCID patients born yearly in the United States and Canada undergo definitive treatment (1, 2). Over the past 10 years, the addition of T-cell receptor excision circle (TREC) assays to the newborn screen has allowed for earlier diagnosis, therapy, and improved survival for patients with SCID (3). With a growing population of post-transplant SCID patients living into adulthood with a wide spectrum of immune functions, it is critical to understand their long-term outcome.

The most common genetic defects resulting in SCID are IL-2 receptor gamma (*IL2RG*) gene variants causing X-linked SCID (X-SCID) (2). This gene encodes the common gamma chain (γc), which is a subunit of the IL-2, IL-4, IL-7, IL-9, IL-15, and IL-21 receptors. X-SCID is typically characterized by the absence of T and NK cells due to ineffective signaling of IL-7 and IL-15, respectively (4). B cells in X-SCID are typically normal in number, but they have an abnormal function due to impaired IL-21 signaling, which is critical for the maturation, differentiation, and activation of the B-cell lineage (4, 5).

The survival rate of patients with SCID who receive HLA-identical transplants is ~90% (3, 6). Long-term survival is affected by the causal genetic defect, donor match, conditioning regimen, age at transplant, and clinical status of the patient prior to transplant. The single most important factor in survival is donor match, with HLA-identical sibling donors providing the best outcomes (3, 7). The majority of deaths result from infections at the time of transplant or within the first year post-transplant, with the second leading causes of death being pulmonary disease or acute respiratory distress syndrome (3, 7, 8). However, sequalae for long-term survivors have not been systematically reported.

## Case Presentation

The patient is a 48-year-old man with a family history that includes the infantile deaths of three maternal uncles. He presented at 5 months of age with recurrent oral thrush and *Pneumocystis jiroveci* pneumonia. He later developed *Candida* esophagitis and failure to thrive. The patient received an unconditioned transplant at 11 months of age in 1971 that comprised whole, unprocessed bone marrow (9). HLA compatibility with his 11-year-old sister, the donor, was determined by HLA serotyping and mixed leukocyte culture. The immediate post-transplant course was complicated by acute Graft vs. Host Disease (GvHD) with rash, fever, hepatosplenomegaly, and respiratory distress that resolved after treatment with prednisone. In the first 10 years post-transplant, he developed chronic dry eyes, a few cases of pneumonia, and one episode of cellulitis. He was lost to follow-up at age 17.

**Figure 1:**
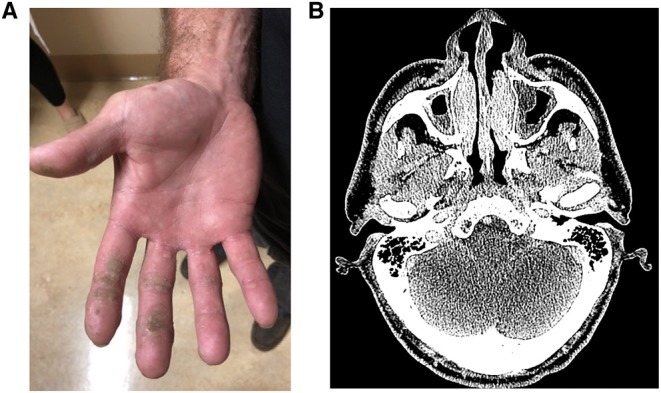
**(A)** Numerous, confluent cutaneous warts on palmar surface of hand. **(B)** CT sinuses showing turbinate edema and maxillary sinus mucosal thickening with layering fluid.

He was referred back to UCLA Immunology three decades later because of the failure of four corneal transplants. The patient reported living a “normal life” working as a plumber. He had never required immunoglobulin (Ig) replacement or nutritional support. He had recurrent dental infections, and one skin carcinoma had been removed. He developed cutaneous, oropharyngeal, and genital warts that relapsed and remitted but had worsened in recent years (Figure 1A). In the prior 5 years, he had three pneumonias, chronic sinusitis (Figure 1B), and a skin infection due to MRSA.

The *IL2RG* gene was sequenced from patient's buccal mucosal cells, and this revealed a hemizygous variant on Chromosome X at location g.70329160 (based on build GRCh37/hg19), NM_000206.2:c.675C>A, resulting in p.Ser225Arg. Cases of X-SCID have been reported due to missense variants of nearby amino acids (R222C, R224W, R226C/H, F227C, and L230P) (10). This variant does not appear in large population databases of healthy subjects (11) and has been identified as pathogenic in one other infant with a T^−^B^+^NK^−^ SCID phenotype (12). Thus, we diagnosed our patient with X-SCID.

Lymphocyte enumeration showed normal numbers of T and B cells but low numbers of NK cells. A predominantly memory phenotype was seen for both CD4^+^ and CD8^+^ T cells (Table 1). TRECs were absent. Lymphocytes responded with modest proliferation after stimulation with mitogens PHA, PWM, and ConA. There was a minimal proliferative response to either tetanus antigen or *Candida*. The response to Staph enterotoxin B stimulation was robust. Foxp3^+^ Tregs comprised 2.7% of circulating CD4^+^ T cells (normal).

**Table 1:** Laboratory results.

**Lymphocyte enumeration**	**Patient (Cells/μL)**	**Reference (Cells/μL)**
CD3+	1,515	603–2,990
CD4+	511	441–2,156
CD8+	1,116	125–1,312
CD56+/CD16+	17	95–640
CD19+	230	107–698
CD4:CD8	0.52	
	**Patient (%)**	**Reference (%)**
Memory B cells (CD27+ of CD19+)	30	6–52
Switched memory B cells (IgD-IgM- of CD19+CD27+)	5	2–26
Immature B cells (CD21 low of CD19+)	8	0–9
CD4 Naïve (CD45RA+CD62L+ of CD4+)	4	26–64
CD4 Effector memory (CD45RA-CD62L- of CD4+)	65	9–24
CD4 Central memory (CD45RA-CD62L+ of CD4+)	26	22–53
CD4 TEMRA (CD45RA+CD62L- of CD4+)	5	0–4
CD8 Naïve (CD45RA+CD62L+ of CD8+)	6	22–71
CD8 Effector memory (CD45RA-CD62L- of CD8+)	42	8–32
CD8+ Central memory (CD45RA-CD62L+ of CD8+)	10	2–21
CD8 TEMRA (CD45RA+CD62L- of CD8+)	41	0–43
**Immunoglobulins**	**Patient**	**Reference**
IgG (mg/dL)	1,370	726–1,521
IgA (mg/dL)	527	87–426
IgM (mg/dL)	292	44–277
IgE (kIU/L)	6	20–100
**Streptococcal pneumonia titers (μg/mL)**	**Pre-pneumovax**	**Post-pneumovax**
Serotype 1 (1)	<0.3	0.3
Serotype 2 (2)	1.6	1.2
Serotype 3 (3)	<0.3	<0.3
Serotype 4 (4)	<0.3	<0.3
Serotype 5 (5)	0.7	0.8
Serotype 8 (6)	0.8	1.1
Serotype 9 (9N)	<0.3	<0.3
Serotype 12 (12F)	2.0	1.1
Serotype 14 (14)	1.4	0.4
Serotype 17 (17F)	<0.3	<0.3
Serotype 19 (19F)	2.6	4.2
Serotype 20 (20)	0.3	0.4
Serotype 22 (22F)	<0.3	<0.3
Serotype 23 (23F)	16.1	2.9
Serotype 26 (6B)	0.7	1.0
**Streptococcal pneumonia titers (μg/mL)**	**Pre-pneumovax**	**Post-pneumovax**
Serotype 34 (10A)	<0.3	<0.3
Serotype 43 (11A)	5.1	<0.3
Serotype 51 (7F)	<0.3	<0.3
Serotype 54 (15B)	<0.3	<0.3
Serotype 56 (18C)	<0.3	<0.3
Serotype 57 (19A)	<0.3	<0.3
Serotype 68 (9V)	<0.3	<0.3
Serotype 70 (33F)	<0.3	<0.3
	26% protective	9% protective
**Other antibody titers and PCR results**	**Result**	**Interpretation**
Tetanus IgG (IU/mL)	3.7	Protective
HiB IgG (μg/mL)	0.1	Not protective
EBV DNA PCR (copies/mL)	Negative	
EBNA-1 IgG	Negative	
EBV-VCA IgM	Negative	
CMV Ab	Positive	
CMV DNA PCR (IU/mL)	Not detected	
HSV 1 type specific IgG (index)	10.03	Positive
HSV 2 type specific IgG (index)	<0.90	Not detected
HSV 1 DNA PCR	Negative	
HSV 2 DNA PCR	Negative	
Poliovirus type I	<1:10	Not detected
Poliovirus type III	<1:10	Not detected
Varicella IgG (IV)	75	Negative
Measles Ab immune status	Negative	

The diversity of the patients' T cells was examined by assessing the frequency of usage of 24 TCR Vβ regions in T cells by flow cytometry. We found 31.1% of his T cells were Vβ3^+^ (normal range 0.6–8.2%), 22.5% of his T cells were Vβ1^+^ (normal range 1.9–4.7%), and 5.7% of his T cells were Vβ16^+^ (normal range 0.5–2.9%), while proportions of 15 other Vβ regions were lower than normal. Thus, while T-cell numbers were normal, the patient's T cells had a diminished repertoire.

*In vitro* assays revealed that NK-cell-dependent cytotoxicity was impaired in the patient. We examined killing after normalizing for numbers of NK cells and offered a range of erythroblastoid target cell concentrations, but killing was <2% and was not accentuated by pre-incubation with the γc cytokines IL-2 or IL-15. This result suggested that his NK cells were poorly functional.

HLA typing was performed by sequencing DNA isolates from the patient's buccal mucosal cells, confirming a 12-out-of-12 match with the donor. To assess engraftment and the origins of specific hematopoietic lineages, we performed chimerism studies on sorted cells by short-tandem repeats. As expected, myeloid cells were entirely from the recipient, and T cells were entirely from the donor. However, B cells were 93% from the recipient and 7% from the donor, and the degree of uncertainty in this test did not preclude the possibility that 100% of the B cells are from the recipient. NK cells were 69% from recipient. To further understand the origins of these cells, we rigorously purified T cells, B cells, and monocytes by flow sorting and analyzed each population with whole exome sequencing. None of the T cells showed rare variants in *IL2RG*, while 100% of the monocytes and B cells showed the pathogenic variant of *IL2RG*. Thus, a somatic reversion mutation was not responsible for the resiliency of the patients' B cells nor were any of the B cells derived from the donor.

Thirty percent of the patient's B cells had a memory phenotype, and 5% were of the switched memory subtype (Table 1). These proportions were normal for his age. His serum immunoglobulin levels were normal to elevated across all isotypes, and he had never been on Ig replacement. B-cell function, as measured by antibody titers, showed measurable titers to tetanus, CMV, and HSV-1 but no protective titers for *Streptococcus pneumoniae*, polio, or HiB. He had absent titers to EBV and HSV-2. Boosting with polysaccharide pneumococcal vaccine resulted in poor responses (Table 1). These studies suggest a specific antibody defect to polysaccharide antigens but otherwise relatively intact B-cell function.

**Figure 2:**
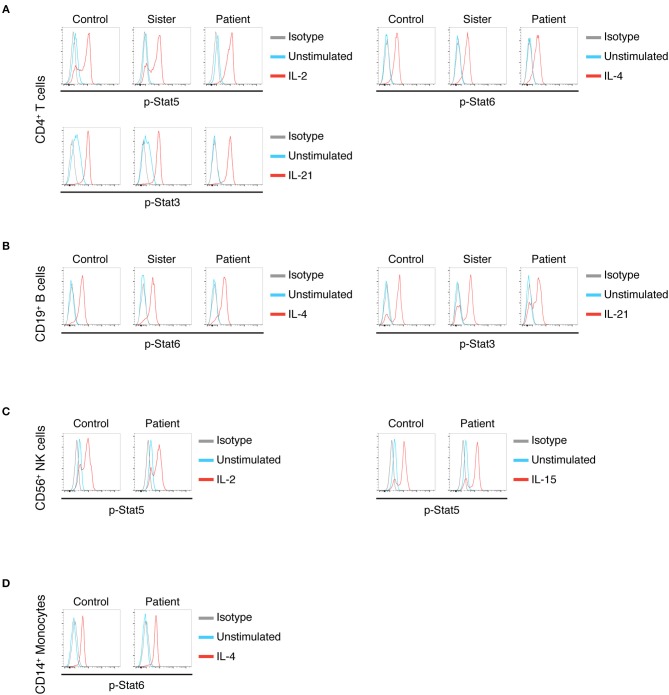
**(A)** IL-2, IL-4, and IL-21 stimulation in CD4^+^ T cells in control, sister (donor) and patient, as measured by pSTAT5, pSTAT6, and pSTAT3, respectively. **(B)** IL-4 and IL-21 stimulation of B cells in control, sister (donor), and patient as measured by pSTAT6 and pSTAT3. **(C)** IL-2 and IL-15 stimulation in NK cells in control and patient as measured by pSTAT5. **(D)** IL-4 stimulation in CD14^+^ monocytes in control and patient as measured by pSTAT6.

To better delineate our patient's B-cell function, we studied the response of his lymphocytes to cytokines that signal through the γc receptor (Figure 2). Typically, post-HSCT X-SCID patients with split chimerism show low-to-normal *in vitro* B-cell responses to stimulation with IL-4 but low-to-absent responses to IL-21 (13). This discrepancy is attributed to the ability of IL-4 to bind and signal through either a receptor comprising γc and IL-4RA or an alternate receptor comprising IL-4RA and IL-13R chains. On the other hand, γc is indispensable for IL-21 signaling (5, 13, 15). We found our patient's T cells showed normal γc signaling (Figure 2A). When we tested B-cell responses to γc-dependent cytokines, we found that B cells stimulated with IL-21 showed normal phosphorylation of STAT3 (Figure 2B). This result suggests that the genomic variant γc, which is present in the B cell population, retains normal IL-21 signaling. Most NK cells showed normal γc signaling (Figure 2C), corresponding to the degree of chimerism. Finally, we found that IL-4 stimulation showed normal phosphorylation of STAT6 in B cells and monocytes.

## Discussion

To our knowledge, this is the longest follow-up of a SCID patient who received an allogeneic transplant. The first SCID transplants were performed in the late 1960s (16, 17), and the few patients who received transplants prior to ours, to the best of our knowledge, have not had immune evaluations beyond three decades post-transplantation (17, 18). Our patient had experienced some complications post-transplant, including acute GvHD, bacterial infections (pneumonia, sinusitis, and cutaneous abscesses), and chronic warts. However, he had not experienced other common complications of post-transplant SCID patients, such as chronic GvHD, severe or opportunistic infections, or autoimmunity, as seen in up to 15% of cases. More importantly, he has not required Ig replacement or nutritional support as required in a subset of post-transplant patients (7, 14, 18, 19).

Warts due to HPV infection typically develop 10 years after a transplant and continue to worsen over time. Warts are especially common post-transplantation in patients bearing pathogenic variants in *IL2RG* and *JAK3* with as many as 26% of X-SCID patients developing warts (8, 18, 20). Defective γc signaling in keratinocytes, particularly through the IL-15 receptor, may also contribute to wart susceptibility by reducing chemokine production and CD4^+^ T cell recruitment (21). As in our patient, low NK cell counts are found in up to 33% of post-transplant patients, and may further contribute to HPV susceptibility (22). Defects in NK cell function also increase risk of viral induced cancers (23, 24). HPV infection, including genital warts, and poor NK killing in our patient put him at high risk for squamous cell carcinoma, highlighting the need for continued monitoring.

Many SCID patients require Ig replacement after transplantation, particularly in X-SCID patients who underwent transplantation without conditioning (8, 14). Whether B-cell chimerism influences B-cell function and the need for Ig replacement is controversial (7, 13, 25). As was the case in our patient, a few post-transplant X-SCID patients without B-cell chimerism have not required Ig replacement and even demonstrated antibody responses to neoantigens such as φX174 (13). The mechanism for adequate host B-cell function in these X-SCID patients is not understood (13).

Long-lived protective antibody responses and the formation of memory B cells are dependent on IL-21 signaling through a normal γc (5, 15, 26). In post-transplant patients, at least 10% of B cells must have adequate IL-21 signaling for B cells to function (5, 15). Our patient's B cells were host-derived and carried the pathogenic genomic variant. Yet, IL-21 signaling was functionally intact, as demonstrated *in vitro* by the normal phosphorylation of STAT3 in response to IL-21 and *in vivo* by normal numbers and proportions of memory B cells, normal levels of all immunoglobulins, and relatively intact antibody responses. We hypothesize that there may be leaky IL-21 function in post-transplant patients who maintain adequate B cell function but lack donor B cells, allowing them to meet this critical 10% functional threshold.

Normal signaling through the IL-21 receptor in B cells bearing a pathogenic γc is novel to our knowledge. However, it is well-known that missense variants in γc can lead to atypical SCID (2). Missense variants in exon 5, where our patient's variant falls, account for 29.4% of known γc mutations. Phenotypes in this region are highly variable based on the variant. If exon 5 variants fall in the highly conserved WSXWS motif, the effect is a severe phenotype (27), whereas other nearby variants can confer differential loss of cytokine signaling. For example, in the nearby p.R222C (4). In these patients, IL-4 signaling was comparable to control, some IL-15 and IL-2 signaling were seen at high agonist concentrations, but IL-21 signaling was absent. As a consequence, intact numbers of NK cells can often be seen in p.R222C (27), and even normal T-cell development has been noted (28). In contrast, our patient's variant resulted in an amino acid substitution 12 amino acids upstream from the WSXWS motif (29) and his B cells showed normal signaling in response to stimulation with IL-21.

Our patient has illustrated the excellent survival of matched sibling donor transplants (3). He has also demonstrated the importance of long-term follow-up, given unknown graft duration, morbidity associated with HPV infection, and concern for malignancy. Overall, survival post-transplantation has improved over time (3). However, the incidence of repeat transplants or gene therapy after initial transplant, has increased in the past 20 years due to reduced intensity conditioning regimens (7). In our patient, the natural aging of the donor T cells and lack of genesis of new T cells suggests that he may benefit from a booster transplant from his sister for his persistent cutaneous warts and recent bacterial infections.

## Summary

In summary, 47 years after the unconditioned bone marrow transplant, our patient was doing relatively well. His T cells were adequate in number, although somewhat oligoclonal. Importantly, they were functionally sufficient, able to maintain adequate immunity against most pathogens, and able to provide B-cell help for T-dependent humoral responses. His ongoing B-cell function was attributable to a combination of close HLA matching to his donor, allowing for effective T-cell help, and a hypomorphic γc mutation that allowed for IL-21 signaling. We speculate that other X-SCID patients with functional host B cells post-transplant may bear similar hypomorphic mutations that allow for functionally relevant cytokine signaling.

## Methods

### Reagents

Several antibodies were used: anti-pStat3 (Tyr705) AF647 (clone 4/P-Stat3), anti-pStat6 (Tyr641) AF647 (clone 18/P-Stat6), anti-pStat5 (Tyr694) AF488 [clone 47/Stat5(pY694)], and Mouse Isotype Control AF488 or AF647 (clone MOPC-173) from BD Biosciences; anti-CD4 AF488 or BV421 (clone OKT4), anti-CD14 BV421 (clone HCD14), anti-CD19 BV421 (clone HIB19), and Human TruStain FcX from Biolegend. All cytokines were from Peprotech: IL-2 (Cat# 200-02), IL-4 (Cat# 200-04), and IL-21 (Cat# 200-21).

### Phospho-Stat Assays

*Stimulation*: 20 μL of 10X cytokines in PBS (or PBS only control) were added to 180 μL of heparinized whole blood in a 5 mL FACS tube to achieve these final concentrations: IL-2 (10 ng/mL), IL-4 (100 ng/mL), or IL-21 (50 ng/mL). Tubes were incubated at 37°C for 20 min, at which point 4 mL of pre-warmed 1X BD Lyse/Fix buffer (Cat# 558049) was added. Cells were fixed for 10 min at 37 °C, centrifuged and washed twice with FACS buffer (1X PBS supplemented with 2% FBS and 1 mM EDTA). *Staining and permeabilization*: Fc receptors were blocked for 5 min at RT, followed by a 20 min stain on ice with anti-CD4 and anti-CD19. Cells were washed with FACS buffer and permeabilized for 30 min on ice with 1 mL BD Phosflow Perm Buffer III (Cat# 558050) that had been pre-cooled to −20°C. After permeabilization, 2 mL FACS buffer was added and the samples were centrifuged. After three additional washes, the cells were stained with anti-pStat antibodies or an isotype control for 30 min at RT. Samples were washed three times and data were collected on a Cytek DxP10 flow cytometer. Data were analyzed with FlowJo software.

## Data Availability Statement

All data for this paper are available in this article/supplementary materials.

## Ethics Statement

The studies involving human participants were reviewed and approved by UCLA Office of the Human Research Protection Program. The patients/participants provided their written informed consent to participate in this study. Written informed consent was obtained from the individual(s) for the publication of any potentially identifiable images or data included in this article.

## Author Contributions

CD, MG-L, and MB provided care. CD wrote the manuscript. All authors edited the manuscript. MB and ES designed the experiments. TT performed the experiments.

### Conflict of Interest

The authors declare that the research was conducted in the absence of any commercial or financial relationships that could be construed as a potential conflict of interest.
